# Thermochromic Polymer Nanocomposites for the Heat Detection System: Recent Progress on Properties, Applications, and Challenges

**DOI:** 10.3390/polym16111545

**Published:** 2024-05-30

**Authors:** A. B. M. Supian, M. R. M. Asyraf, Agusril Syamsir, M. I. Najeeb, Abdulrahman Alhayek, Rayeh Nasr Al-Dala’ien, Gunasilan Manar, A. Atiqah

**Affiliations:** 1 Institute of Energy Infrastructure, Universiti Tenaga Nasional, Jalan IKRAM-UNITEN, Kajang 43000, Selangor, Malaysia; 2 Centre for Defence Research and Technology (CODRAT), Universiti Pertahanan National Malaysia, Kem Perdana Sungai Besi, Kuala Lumpur 57000, Malaysia; gunasilan@upnm.edu.my; 3 Engineering Design Research Group (EDRG), Faculty of Mechanical Engineering, Universiti Teknologi Malaysia, Johor Bahru 81310, Johor, Malaysia; 4 Centre for Advanced Composite Materials (CACM), Universiti Teknologi Malaysia, Johor Bahru 81310, Johor, Malaysia; 5 Civil Engineering Department, Universiti Tenaga Nasional, Jalan IKRAM-UNITEN, Kajang 43000, Selangor, Malaysia; rahman.hayek@gmail.com (A.A.);; 6 Institute of Microengineering and Nanoelectronics, Universiti Kebangsaan Malaysia, Bangi 43600, Selangor, Malaysia

**Keywords:** reversible thermochromic, thermochromism, polymer nanocomposites, heat detection sensing, colour-changing materials

## Abstract

Reversible thermochromic polymers have emerged as compelling candidates in recent years, captivating attention for their application in heat detection systems. This comprehensive review navigates through the multifaceted landscape, intricately exploring both the virtues and hurdles inherent in their integration within these systems. Their innate capacity to change colour in response to temperature fluctuations renders reversible thermochromic nanocomposites promising assets for heat detection technologies. However, despite their inherent potential, certain barriers hinder their widespread adoption. Factors such as a restricted colour spectrum, reliance on external triggers, and cost considerations have restrained their pervasive use. For instance, these polymer-based materials exhibit utility in the domain of building insulation, where their colour-changing ability serves as a beacon, flagging areas of heat loss or inadequate insulation, thus alerting building managers and homeowners to potential energy inefficiencies. Nevertheless, the limited range of discernible colours may impede precise temperature differentiation. Additionally, dependency on external stimuli, such as electricity or UV light, can complicate implementation and inflate costs. Realising the full potential of these polymer-based materials in heat detection systems necessitates addressing these challenges head-on. Continuous research endeavours aimed at augmenting colour diversity and diminishing reliance on external stimuli offer promising avenues to enhance their efficacy. Hence, this review aims to delve into the intricate nuances surrounding reversible thermochromic nanocomposites, highlighting their transformative potential in heat detection and sensing. By exploring their mechanisms, properties, and current applications, this manuscript endeavours to shed light on their significance, providing insights crucial for further research and potential applications.

## 1. Introduction

**Figure 1:**
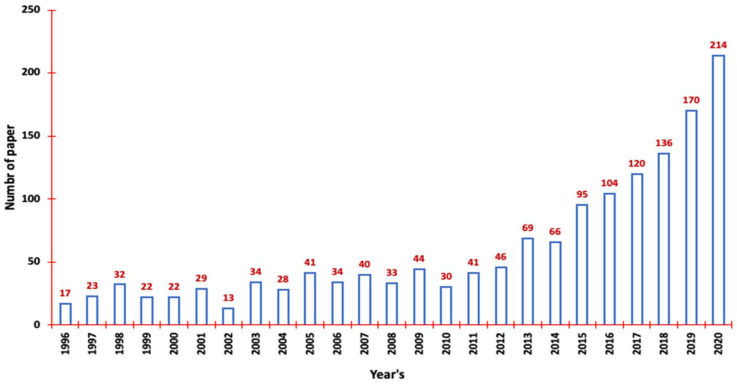

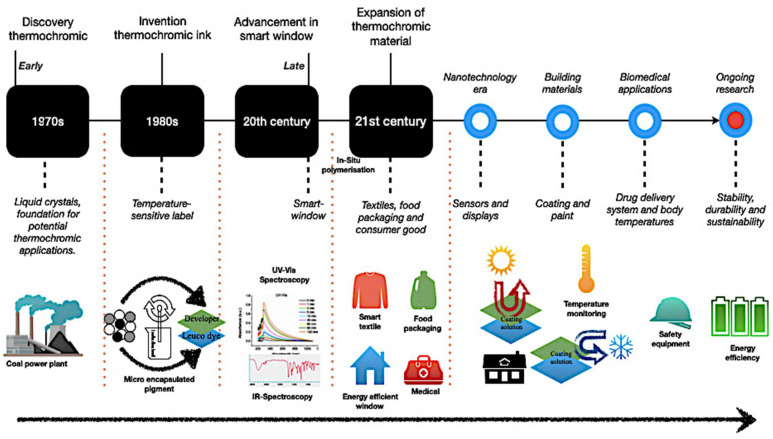
The milestone and history of reversible thermochromic materials with the development of reversible thermochromic materials in various technological applications with various analysed papers published in the different time regimes [24,25,32,40].

Chromic materials, capable of dynamic colour changes, represent a captivating realm where hues respond to external stimuli. These materials span diverse categories, including photochromic, thermochromic, and electrochromic variants, demonstrating versatile optical properties and adaptability to colour shifts under different triggers [1,2,3,4,5,6,7,8,9,10,11,12,13]. Table 1 shows that recent strides in thermochromic polymer nanocomposites have notably fortified their stability and versatility, auguring significant implications across multiple industries [10,14,15,16,17,18,19,20,21,22,23]. The evolution of thermochromic materials, depicted in Figure 1, began in the 1970s, primarily focusing on colour transitions linked to temperature [24,25]. Gradually, research progressed from mere novelties to nanostructured variants, enabling precise colour modulation for sensors, displays, and eco-friendly coatings. These materials now hold potential for energy-efficient building coatings and drug delivery systems, highlighting their role in sustainability and energy conservation [26,27,28]. Their growing relevance aligns with global energy conservation initiatives. For example, “cool roofs,” aiming to minimise solar energy absorption and surface temperatures, showcase the materials’ role in enhancing energy efficiency, especially in warmer regions. Given the substantial energy consumption by the building and automotive industries, these materials significantly impact urban environments, influencing energy use and indoor air quality [29,30,31,32,33,34].

Understanding and employing reversible thermochromic materials requires a comprehensive assessment across structural, optical, thermal, and mechanical aspects. Techniques like UV–Vis spectroscopy, infrared spectroscopy, and Raman spectroscopy help analyse critical parameters, offering insights into their responsiveness [35,36,37,38,39,40,41]. Recent advancements show promise by enabling colour changes with temperature shifts across various sectors. Improved manufacturing methods aim to enhance the efficiency of temperature-responsive windows while reducing energy consumption and promoting eco-friendly waste management [42,43,44,45]. These methods involve molecular changes, phase transitions, and alterations in nanoparticles or molecular structures induced by temperature variations. Some materials use specific dyes or pigments to achieve colour changes, with various techniques like in situ polymerization and nanoparticle encapsulation developed for specific applications such as temperature sensors and smart coatings [19,34,46,47,48,49,50,51,52,53].

Addressing challenges like durability in reversible thermochromic coatings demands robust solutions. Implementing stringent manufacturing processes and protective layers has been pivotal [3,54,55,56,57,58,59,60,61,62,63]. Recent research explores theoretical frameworks, nanocomposite polymer films, photonic fibres, and smart polymers to bolster functionalities and expand usage [10,46,64,65,66]. Reversible thermochromic materials’ adaptability makes them powerful tools across industries, especially in sensor-based heat detection devices.

In construction, integrating these materials into thermal imaging systems aids in the real-time identification of heat loss or inadequate insulation. This not only flags energy inefficiencies but also prompts swift rectification measures, enhancing energy conservation. Within the automotive sector, applying reversible thermochromic coatings to engine components or electrical systems creates temperature sensors, critical for monitoring thermal conditions. These sensors prevent overheating risks, ensuring better safety and durability and averting potential breakdowns or accidents. In healthcare, using these materials in smart bandages or medical devices enables non-invasive body temperature monitoring. Such applications help assess fever or inflammation, aiding timely diagnoses and patient care.

These applications demonstrate the diverse potential of reversible thermochromic materials in sensor-driven heat detection devices across industries. Their adaptability, reliability, and responsiveness position them as valuable tools in temperature monitoring, energy conservation, and safety enhancement. Despite challenges, they offer cost-effective, real-time solutions for thermal management and temperature monitoring, promising transformative impacts in various fields. This paper reviews reversible thermochromic nanocomposites for sensor applications, covering classification, mechanisms, properties, and current prospects, providing insights for research and future applications.

## 2. Reversible Thermochromic

### 2.1. Fundamental of the Reversible Thermochromism

**Figure 2:**
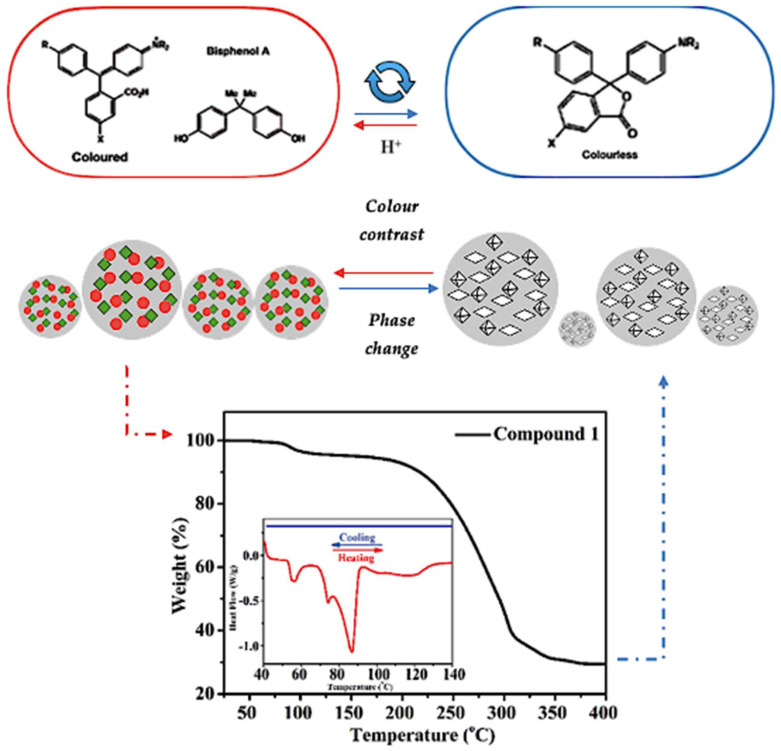
Schematic relationship between temperature and colour change in a specific reversible composite organic thermochromic pigment [19,52,67,68,69].

Reversible thermochromism is a fascinating property seen in certain materials that change colour as temperatures vary. Comprehending this phenomenon is crucial for tailoring materials with temperature-dependent colour shifts. When exposed to different temperatures, reversible thermochromic materials change colour reversibly, returning to their original hue when the temperature normalizes. This effect occurs due to molecular rearrangements, energy level transitions, or changes in particle size affecting light absorption, reflection, or scattering, leading to observable colour changes. Mechanisms behind reversible thermochromism, like conformational shifts or reversible chemical reactions, vary per material. Incorporating specific additives or nanoparticles with temperature-dependent optical traits enables controlled colour shifts (see Figure 2). This foundational understanding is vital for crafting materials with customised thermochromic properties, expanding their application in diverse fields [15,22,23]. Otherwise, Figure 2 depicts the thermogravimetry-differential scanning calorimetry (TG-DSC) data that can guide spectroscopic analysis by pinpointing relevant temperature ranges for detailed spectroscopic measurements. This targeted approach enhances the efficiency and informativeness of data collection during spectroscopic studies.

Recent research conducted by Zhang et al. [70] sheds light on polydiacetylene-based thermochromic materials and their functioning. Their study highlighted that temperature-induced structural changes affected the material’s core structure, altering electron distribution and, consequently, the absorption spectrum. This caused a transition in colour, shifting from red to blue as temperatures increased. Additionally, transitions between solid, liquid, or gaseous states affected how these materials interacted with light, further influencing colour changes. Reversible thermochromic materials can also change colour through reversible chemical reactions and by adding thermochromic components. The degree of colour change within specific temperature ranges depends on temperature sensitivity, while chemical components like chromophores and additives impact light absorption and reflection.

Environmental elements like humidity, pressure, and light exposure significantly affect observed colour shifts. Liu et al. [71] research highlighted how varying humidity levels impacted colour transition temperatures in smart thermochromic coatings. Moreover, controlling heating and cooling rates allows for manipulating colour changes. Recent advancements by Wang et al. [72] showed how adjusting temperature change rates affected phase transition kinetics, enabling customised colour-switching speeds in thermochromic materials, and paving the way for adaptable applications in smart sensors and displays.

### 2.2. Thermochromic Mechanism and Classification

**Figure 3:**
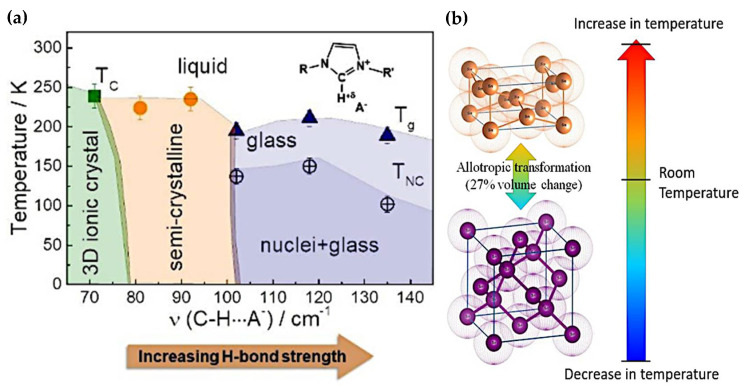
Dynamic correlation of structural phase transition of thermochromic material discoloration due to intermolecular mechanisms in thermochromic during crystalline vs. glass synthesis transformation [85,86]. (**a**) Phase diagram of imidazole-based ionic liquids (Im-ILs) showing the gradual loss of 3D crystalline order with increasing temperature. (**b**) Tin’s electron configuration change alters the crystal lattice due to a temperature change.

**Figure 4:**
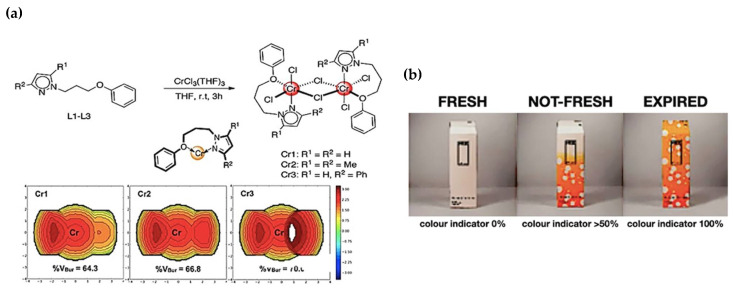
Diagram illustrating the ligand geometry mechanism in chromium-based materials. (**a**) Phenyl ether-pyrazolyl [N,O] ligands as catalysts for the oligo and polymerization of ethylene [96]. (**b**) A milk carton packaging demonstrating the colour change as the expiration date approaches.

**Figure 5:**
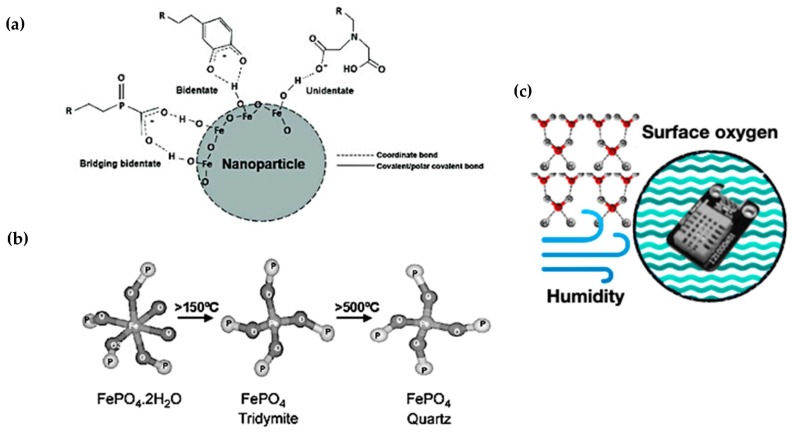
Schematic diagram illustrating the coordination number mechanism in thermochromic materials. (**a**) Coordination bonding between chelating agents and the hydrogens of the surface −OH (hydroxyl) groups of a metal oxide nanoparticle [98]. (**b**) The local structural models employing FePO_4_•2H_2_O by different coordination number and crystal water content [99], (**c**) Humidity sensing device [100].

**Figure 6:**
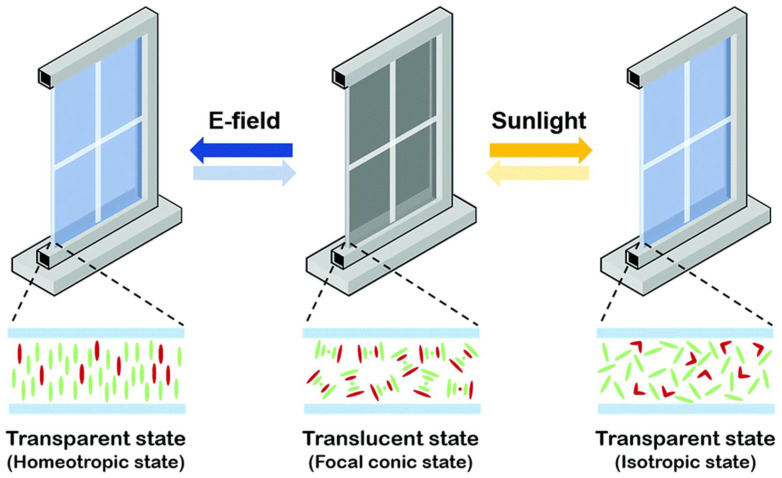
Schematic representation of revisable liquid crystal thermochromic material on smart window using a push–pull chemical compound of functional group R−N = N−R′ [101,102].

Reversible thermochromic materials display intriguing colour changes affected by temperature, composition, and environmental factors [73]. These alterations occur due to changes in their molecular structure when temperatures shift, affecting how they appear visually. Table 2 summarises recent studies on these materials, categorising them based on different triggers, which find applications in devices like heat chambers, electrical components, and transformers [74,75,76]. Understanding the mechanisms behind thermochromism is crucial to grasping how these materials change colour. Various mechanisms are involved, influenced by specific factors, contributing to colour shifts and reversibility. This section aims to explore these fundamental mechanisms, depicted in Figure 3, Figure 4, Figure 5 and Figure 6, through diverse experimental techniques. Table 3 further summarises these mechanisms, allowing tailored materials for applications ranging from smart coatings to industrial monitoring and making them versatile across industries.

a. Crystal transition mechanism

The fascinating phenomenon of thermochromism, especially notable in metal ion compounds, involves crystal transitions triggering colour changes with temperature variations. Understanding the dynamic correlation between structural phase transitions and discolouration in thermochromic materials (Figure 3a), particularly during crystalline versus glass synthesis transformations, elucidates complex intermolecular pathways (Figure 3b). During crystalline synthesis, thermochromic compounds exhibit highly organised molecular structures. At lower temperatures, these structures remain stable, displaying a distinct colour attributed to crystal lattice electrical transitions or absorption processes. However, increased temperature leads to lattice vibrations that disrupt the crystal structure, affecting electron energy levels and altering the absorption spectrum and colour. This dynamic interaction between molecular configurations and thermal energy underlies thermochromism in crystalline materials.

In contrast, glass synthesis results in amorphous or disordered thermochromic materials lacking a long-range structured crystal lattice. Molecular mobility rises with temperature, influencing interactions between neighbouring molecules and, thus, changing the material’s electrical and optical characteristics, leading to colour changes. Various intermolecular mechanisms contribute to the dynamic relationship between structural phase transitions and discolouration in thermochromic materials. Crystalline synthesis-induced lattice disruptions alter the electrical environment, modifying the absorption spectrum and colour with temperature. Conversely, in glass synthesis, changes in molecular mobility and local environments influence optical characteristics, causing colour shifts without structural alterations.

For instance, compounds like Cu_2_HgI_4_ and Ag_2_HgI_4_ change colour as their structure shifts from tetrahedral to cubic when heated. Understanding this crystal transition is vital for real-world applications like architecture, where reversible thermochromic materials in building coatings identify thermal inefficiencies, aiding in energy conservation and cost-effective heating [87,88]. In automotive paint, thermochromic coatings signal potential overheating in engines or electrical systems, preventing failures and ensuring safety [84,89]. Moreover, in healthcare, thermochromic materials are explored for body temperature sensors, providing non-invasive monitoring and aiding in prompt medical interventions, especially for fever or hypothermia [85,90]. These applications highlight the adaptability and versatility of these materials across industries, showcasing their potential for diverse needs, from energy efficiency to health monitoring.

b. Ligand geometry mechanism

Thermochromism, observed in inorganic materials like those containing Cr^3+^ ions, occurs when the molecular arrangement changes with temperature [91,92]. For instance, materials with Cr^3+^ ions shift colours by adjusting their structure when heated, whereas the ions around the central ion are altered due to an expansion in the crystal lattice’s octahedral structure. This change in ion arrangement inside the crystal influences the material’s colour (Figure 4a) [93,96]. In real-world applications, inorganic thermochromic materials play a role in smart packaging for perishable goods, as seen in Figure 4b, with milk carton packaging changing colour near its expiration date. These smart packages, integrated with thermochromic materials, react to temperature changes caused by spoilage. This colour shift signals changes in product freshness or environmental temperature. The mechanisms behind these materials influence the packaging’s colour, serving as a warning to consumers or handlers about potential temperature-related issues during storage or transport.

c. Coordination number mechanism

The concept of coordination number, essential in complex compounds, plays a crucial role in thermochromism, especially in inorganic salts like nickel and cobalt salts, often containing crystal water [46]. Take iron phosphate (FePO_4_•2H_2_O) as an example. It exhibits two structural forms—orthogonal and monoclinic—each linked to distinct coordination numbers. The presence or loss of crystal water profoundly affects its colour. With crystal water, it is a light yellowish–white powder; without, it turns yellow–white [76]. Materials sensitive to humidity changes, altering colour based on moisture levels, can power humidity-sensing devices. Similarly, smart packaging employing these materials could signal temperature or humidity changes in food storage, ensuring food quality [97].

d. Liquid crystal mechanism

Reversible liquid crystal thermochromic materials, particularly the cholesteric type, exhibit a unique mechanism that guides their colour changes. Figure 6 depicts their structure as a spiral arrangement with a variable pitch, defining how tightly the spiral turns. As temperature shifts, this spiral’s pitch alters, changing the light wavelength the material reflects and absorbs. This shift in light wavelength affects its intensity, resulting in colour changes [95]. Researchers can customise these materials by tweaking the spiral pitch response to temperature changes and tailoring colour behaviour to specific needs. Additionally, these materials serve as temperature-sensitive indicators in industries, helping to prevent overheating or machinery failures.

### 2.3. Synthesis Techniques for Reversible Thermochromic

Creating reversible thermochromic materials involves methods crucial for their wide application in various fields. These methods aim to make these materials more adaptable, responsive, and energy-efficient across different uses. Techniques like in situ polymerization, solution mixing, melt blending, nanoparticle encapsulation, and coating/impregnation are pivotal for tailoring thermochromic properties [74,75,103,104]. Notable advancements in various deposition techniques include the chemical deposition method and nanocomposite coatings and impregnation method to synthesize and manufacture thermochromic composite and facilitate nanocomposite solar energy transmission based on temperature changes, as developed by Zhang et al. [105], and polymer nanocomposites created by Abdellaoui et al. [46], enabling responsive sensors and versatile materials. In addition, the research by Vaghasiya et al. [106] delved into the integration of various technique (i.e., coating/impregnation and in situ polymerization) to produce wearable thermochromic sensors integrated into flexible patches for health monitoring.

However, challenges persist, such as ensuring consistent exposure and durability; therefore, future studies aim to address these hurdles by enhancing response times, achieving better exposure on complex surfaces, improving durability, and expanding application in various environments. Thus, a combination synthesis technique is used to explore new materials and integrate thermochromic materials into wearable devices that shows promise for future research, as highlighted by Hakami et al. [107]. In addition, Table 4 summarises the schematic of various synthesis techniques used for tailored thermochromic properties, reflecting their potential applications and future research directions.

### 2.4. Characterization of Reversible Thermochromic Materials

Understanding reversible thermochromic materials is vital for their diverse applications in smart windows, energy-saving coatings, and heat detection devices. Table 5 and Table 6 encapsulate a spectrum of characterization techniques utilised to scrutinise these materials. Temperature control plays a pivotal role in spectroscopic and microscopic techniques used to study thermochromic materials. Spectroscopic methods like FTIR and UV–Vis spectroscopy rely on accurately controlling temperature to assess molecular vibrations and absorption spectra as a function of temperature [126]. By monitoring spectral changes at different temperatures, researchers could understand how structural adjustments correlate with colour variations in thermochromic materials, providing insights into their optical behaviour [66].

Similarly, microscopic techniques such as SEM and TEM benefit from temperature control to observe morphological changes and colour variations in high-resolution images [77]. By imaging samples at controlled temperatures, researchers can analyse how temperature influences particle distribution and surface features, facilitating a comprehensive understanding of thermochromic materials’ behaviour [127]. Overall, temperature control enables researchers to unravel the dynamic interplay between temperature, molecular structure, and optical properties in thermochromic materials, crucial for their development and optimization for various applications.

Thermal analysis methods like DSC and TGA measure heat flow, phase transitions, enthalpy changes, and thermal stability, offering crucial insights into material compatibility at various temperatures. dynamic mechanical analysis (DMA) evaluates viscoelastic behaviour, glass transition temperatures, and mechanical stability under different conditions, aiding in understanding material responses in diverse scenarios. Mechanical testing, especially tensile testing, assesses material strength, flexibility, and overall performance, which are essential for evaluating their robustness and mechanical behaviour. Surface analysis through techniques like AFM and XPS investigates surface morphology, roughness, and chemical composition, vital for assessing long-term stability and colour-changing capabilities.

These characterization methods are indispensable for understanding the mechanical, thermal, optical, structural, and surface characteristics of reversible thermochromic materials. They significantly contribute to their analysis and potential applications, ranging from enhancing energy efficiency to tailoring materials for diverse sectors. Future research aims to refine these methods to delve deeper into the intricate behaviour of these materials, optimising their functionality across applications and enhancing adaptability, durability, and responsiveness.

### 2.5. Advance Properties of Reversible Thermochromic Materials

The realm of smart materials introduces an exciting paradigm of adaptability within varying environmental conditions [166]. Among these, thermochromic nanocomposites stand out due to their captivating optical and thermal attributes, making them a focal point of scientific inquiry. This section delves into the diverse techniques employed for assessing these materials (Table 7), showcasing their properties through specific characterization methods.

**Figure 7:**
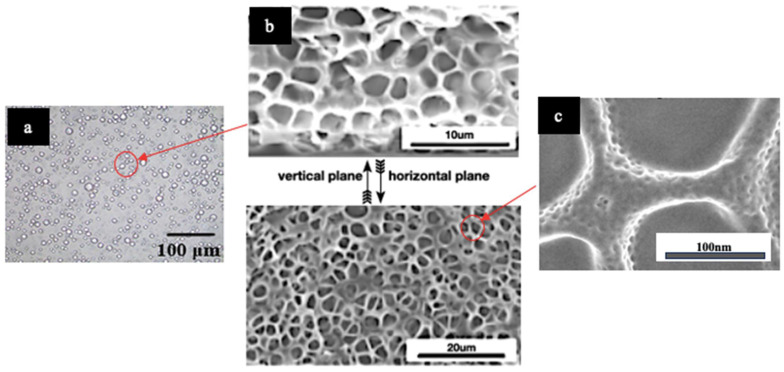
Optical microscopy SEM image of the polymer microstructures of the films: (**a**) cholesteric liquid crystals (CLC) microcapsule, (**b**) polymer microstructures for the as-made films from horizontal and cross-sectional perspectives, and (**c**) cholesteric liquid crystals (CLC) shell thickness [4,6,171,174].

**Figure 8:**
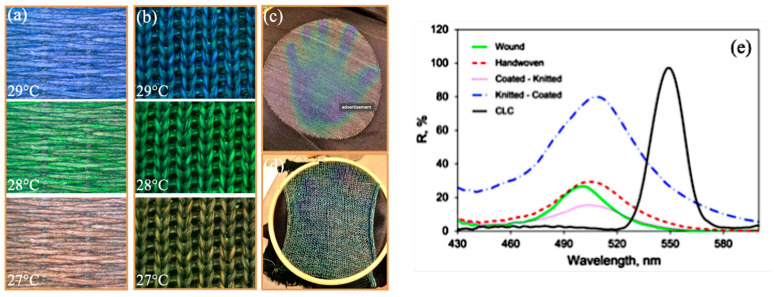
Illustration of thermochromic behaviour in monofilament fibres/cholesteric liquid crystalline composites [4,172]. (**a**) hand woven knitted, (**b**) fabric coated polyester yarns, (**c**) machine knitted fabric, (**d**) handknitted fabric, (**e**) comparison of the reflectance of coated yarn and fabric.

The study by Sun et al. [171] involved the production of microcapsules from cholesteric liquid crystals (CLCs) using the complex coacervation technique. This intricate process, encompassing emulsification, coacervation, and crosslinking, demonstrated remarkable success. The SEM and TEM analyses confirmed the effective encapsulation of CLC, showcasing a particle diameter of about 3–5 µm with uniformed porous structure (Figure 7). Meanwhile, the investigation by Agra-Kooijman et al. [172] utilised polyester filaments pre-coated with thermochromic liquid crystal (TLC) ink to create breathable thermochromic textiles, enhancing their versatility [173]. The combination of pre-coated yarns was selected to achieve the desired thermochromic properties. The resulting knitted and handwoven fabric samples exhibited excellent reversible colour changes from red to blue as the temperature increased from 26 to 32 °C (Figure 8), demonstrating their potential for adaptive applications, consistent with the properties of the TLC ink used.

Furthermore, the innovative approach by Yunxiang Chen et al. [175] involved a novel thermochromic nanocomposite comprising the ionic liquid Nickel–Bromine–Ionic Liquid (Ni-Br-IL) alongside vanadium oxide (VO_2_) nanoparticles. Advanced analyses revealed significant enhancements in thermochromic characteristics, promising applications in smart windows [176]. Therefore, all above discussion studies elucidate the evolving landscape of thermochromic materials; the showcased results not only affirm successful encapsulation and improved thermochromic properties but also hint at prospective applications in diverse fields, especially in smart windows and adaptable materials. The ongoing exploration in this domain paves the way for further research avenues, delving deeper into material synthesis, characterization, and application development for a myriad of industries.

## 3. Application in Heat Detection Systems

The exploration and enhancement of heat-sensing systems utilizing reversible thermochromic materials is a critical frontier in bolstering their operational efficacy [102,177,178,179]. This research phase delves into the nuanced responses of these materials to temperature fluctuations, a cornerstone for advancing efficient heat detection systems. Notably, vanadium oxide, a prevalent component in smart windows, has showcased a noteworthy 9.4% reduction in energy consumption compared to traditional methods, as exemplified by Ye et al. [180]. Assessments extend to diverse environmental conditions such as humidity, UV exposure, and pressure, determining the materials’ adaptability and resilience. Moreover, the seamless integration of thermochromic materials with substrates and coatings significantly amplifies overall system efficiency [181,182,183].

The multifaceted applications of thermochromic nanomaterials are succinctly encapsulated in Table 8, where it comprehensively outlines their varied roles, developmental trajectories, performance attributes, and corresponding references, underscoring their wide-ranging potential across diverse industries and applications.

### 3.1. Thermochromic Materials in Fire Alarm System Applications

**Figure 9:**
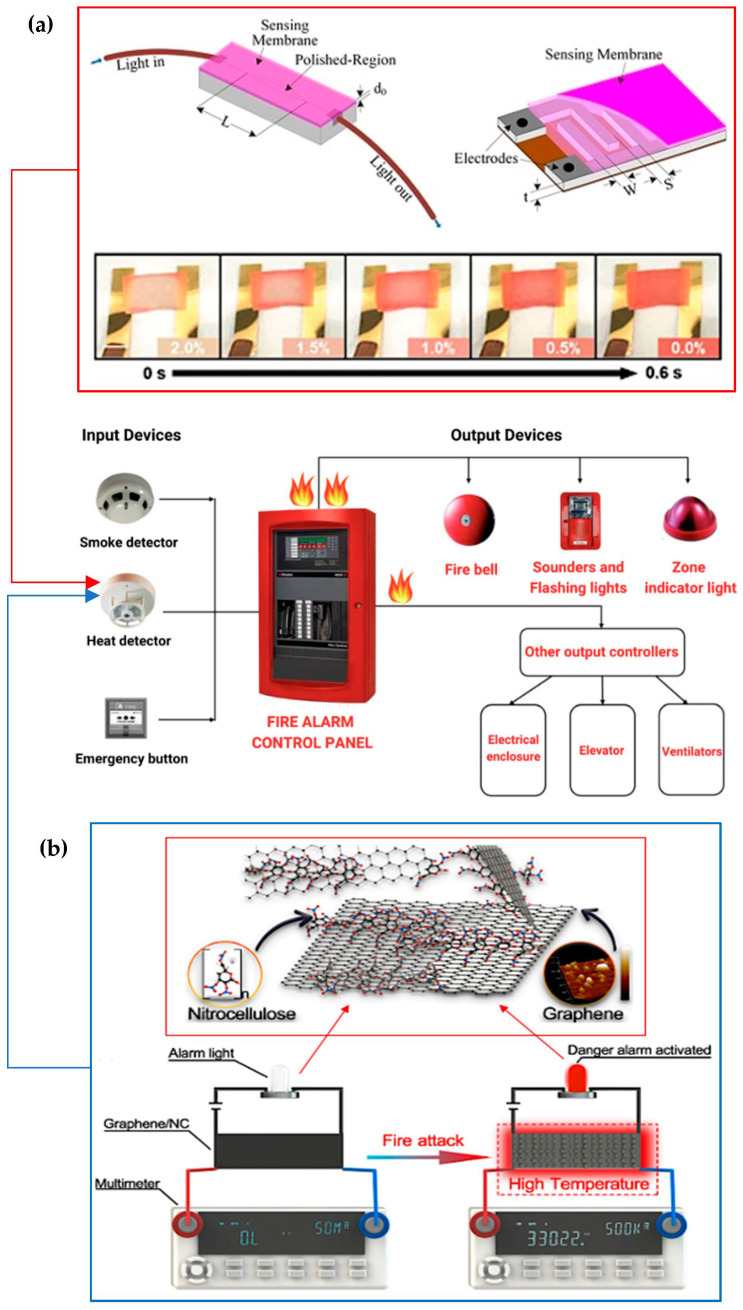
Schematic representation illustrating heat detection with thermochromic material for fire safety systems. (**a**) Thermochromic luminous membrane—effectiveness in high-temperature filtering and fire alarm systems [174]. (**b**) Enhancing fire safety systems rapid response—capabilities of graphene oxide-based fire alarms [177,192].

The significance of smart materials, particularly thermochromic materials in enhancing fire alarm systems, is emphasised by their pivotal role in ensuring safety and mitigating fire-related risks. Notably, previous studies unveil innovative applications and novel material compositions that contribute to advancing fire safety technologies and chemical engineering paradigms towards a more secure, energy-efficient, and environmentally conscious future. Figure 9 shows a schematic heat detection system using a thermochromic membrane material [191] and future research material synthesis of graphene oxide with multifunctional sensor development, as well as integration with intelligent systems for more robust and adaptive fire safety solutions. These advancements underscore nanomaterials’ potential in fire safety engineering. Future efforts in scalable, eco-friendly manufacturing, and durability assessments will shape the evolution of fire safety and chemical engineering.

### 3.2. Coating and Smart Window Systems Applications

Thermochromic materials show potential for energy-efficient smart windows and thermoregulation applications, pivotal for reducing carbon emissions in buildings. Table 9 outlines the various mechanical properties of commonly used thermochromic materials in smart window applications. Liquid crystal polymers, characterised by their liquid crystal structures, exhibit a reorientation temperature range (−20 °C to 50 °C) in thermochromic windows. In the sector of smart windows, a comparison between various thermochromic materials is pivotal, especially concerning their energy-saving potential. Among these materials, vanadium dioxide (VO_2_) coatings have garnered attention due to their cost-effectiveness and scalability for large-scale applications [193]. For instance, Louloudakis et al. [176,194] has demonstrated that VO_2_ coatings exhibit a notable thermochromic transition at low temperature, shifting from a transparent to a reflective state upon heating. It is shown that the precursor flow rate is not the critical factor to influence the thermochromic characteristics of the coatings. Hence, this transition effectively regulates solar heat gain, thereby reducing the need for artificial cooling in buildings [195].

In this case, Arnaoutakis and Katsaprakakis [196] have underscored the significant energy-saving benefits of incorporating VO_2_ coatings into windows. For instance, investigations into energy consumption for heating and cooling in buildings equipped with VO_2_-coated smart windows have revealed substantial reductions compared to conventional windows. The dynamic response of VO_2_ coatings to environmental conditions allows for efficient energy usage, contributing to improved building sustainability and occupant comfort. Ongoing advancements in fabrication techniques and material engineering further enhance the appeal of VO_2_ coatings for widespread adoption in energy-efficient building designs. These findings underscore the potential of VO_2_ coatings as state-of-the-art thermochromic materials for smart windows, offering compelling energy-saving solutions for architectural applications.

**Figure 10:**
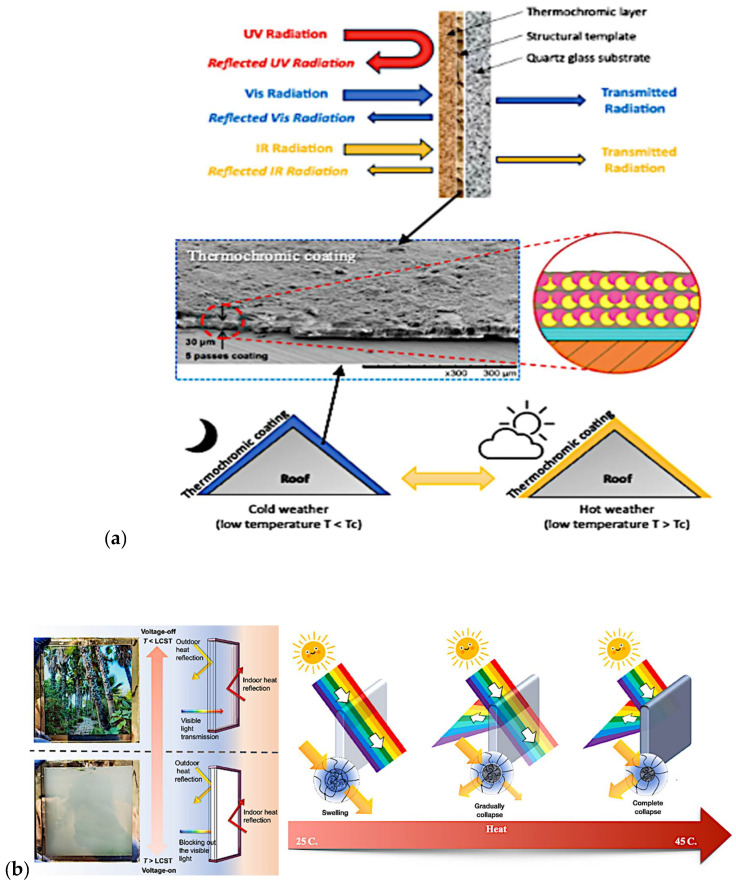
Illustrates the innovation in coating and smart windows. (**a**) Thermochromic coatings for energy-efficient smart buildings and roofs: schematic illustration and scanning electron microscopy (SEM) image of temperature-sensitive paint (TSP) with coating layer schematic [84,197,198]. (**b**) Microgel-based systems intelligently adjust sunlight penetration, and user-controlled windows provide privacy [83,199,200,201].

In summary, this reorientation alters their optical properties, influencing the window’s behaviour and the change in phase or properties of the microcapsule contents due to temperature directly influences the materials’ light transmission properties, affecting their optical characteristics. Furthermore, Figure 10 displays user-controlled smart windows with transparent heaters (smart-shield hydrogel), ensuring privacy and reducing heating and energy saving. These advancements underline the need for thermochromic materials with enhanced mechanical properties, durability, flexibility, and longevity, aligning them with eco-friendly architectural solutions.

**Table 9:** Mechanical properties of the common thermochromic material in smart window.

Material	Description	Type of Smart Window Mechanism	Important Parameter/Value	References
Liquid Crystal Polymers	Polymer Materials with Liquid Crystal Structures	Thermochromic Windows—liquid crystals in the polymer reorient with temperature changes, altering optical properties.	Reorientation temperature range (−20 °C to 50 °C)	[60,62]
Hydrogels	Water-based materials with thermochromic properties	Smart Windows—swelling and shrinking of the hydrogel matrix with temperature variation causes optical changes.	Temperature-induced matrix swelling ratio (20% to 150%)	[63,202,203,204]
Nanoparticles	Tiny particles with reversible thermochromism	Nanoparticle Windows—nanoparticles change their arrangement or properties in response to temperature, affecting light interaction.	Temperature-triggered nanoparticle aggregation temperature (50 °C)	[112,126,144,205]
Microcapsules	Tiny capsules containing thermochromic substances	Microcapsule Windows—the contents of microcapsules change phase or properties with temperature, influencing light transmission.	Phase transition temperature (40 °C to 70 °C)	[113,154]
Polymer-Based Materials	Polymers engineered to exhibit thermochromism	Polymer Smart Windows—the polymer matrix undergoes structural changes with temperature, altering optical characteristics.	Mechanical flexibility rating (excellent, good, fair, or poor)	[32,49,142,155]
Vanadium Dioxide (VO_2_)	Inorganic compound known for its thermochromic properties.	VO_2_ Smart Windows—VO_2_ undergoes a semiconductor-to-metal transition at a critical temperature, affecting its optical properties.	Critical transition temperature (68 °C)	[112,126,147,205]
Liquid Crystal Mixtures	Mixtures of liquid crystals with thermochromic behaviour	Liquid Crystal Windows—liquid crystals in the mixture change orientation and optical properties as temperature varies.	Temperature range for liquid crystal reorientation (25 °C to 60 °C)	[46,64,156]

### 3.3. Food Packaging Application

The role of thermochromic materials in ensuring food safety, quality, and compliance in processing and packaging, characterized by their reversible thermochromic materials, hinges on assessing their colour change precision amid temperature shifts [103]. The use of thermochromic materials in food packaging has notably enhanced food safety and consumer interaction. Chowdhury et al. [81] explored photochromic and thermochromic colourants in packaging, enhancing quality control and consumer interaction. Similarly, Liu et al. [206] introduced non-toxic microcapsules housing thermochromic materials in ink and film, broadening their potential in food packaging. Thus, Table 10 outlines the thermochromic materials and their uses, benefits, and challenges in food packaging applications.

**Figure 11:**
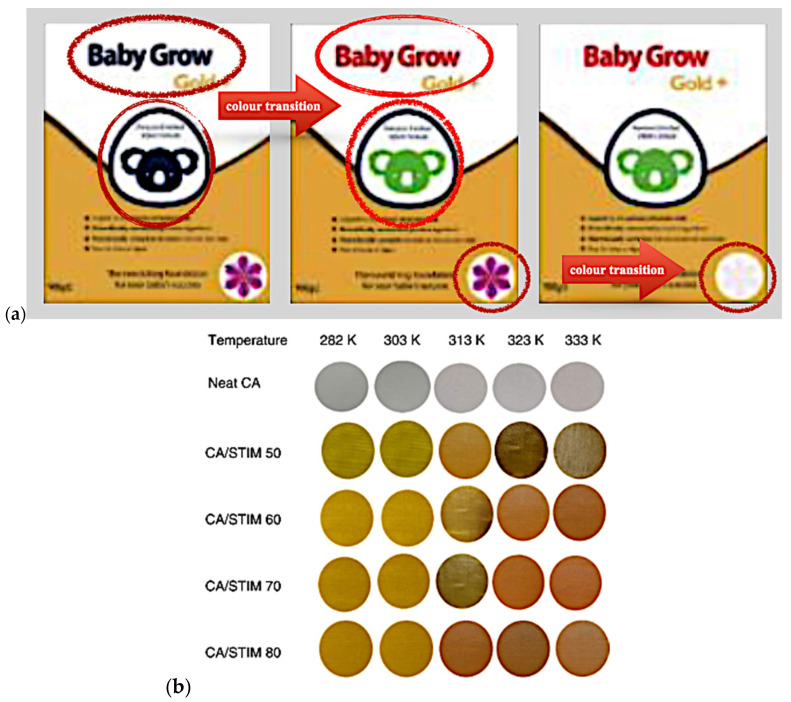
Recent advances in smart packaging technologies. (**a**) Packaging label colour transition due to time periodic monitoring [146]. (**b**) The transition of cellulose acetate and silver tetraiodomercurate-coated labels when exposed to various temperature [207].

Additionally, Figure 11 showcases recent advancements in smart packaging technologies. The periodic transition of packaging labels [146] illustrates time-based monitoring, revealing changes over time. Similarly, the colour transition of cellulose acetate and silver tetraiodomercurate-coated labels with varying temperatures [207] demonstrates visual temperature indication, highlighting the practical application of thermochromic materials in response to diverse thermal conditions. These advancements highlight the importance of thermochromic materials in smart packaging, offering dynamic feedback on temperature changes and time monitoring. Future research could focus on enhancing the durability, adhesion, and safety of these materials for food contact and exploring innovative encapsulation techniques for wider applications in food packaging, further ensuring improved food safety and quality.

### 3.4. Industrial Equipment and Manufacturing

Thermochromic materials play a pivotal role in industrial settings, enabling effective temperature monitoring and process optimization. Techniques like infrared and thermal imaging are key for characterising these materials, offering visual insights into temperature gradients, and identifying equipment hotspots [81,105]. However, ensuring the durability and stability of these materials under demanding industrial conditions is crucial. It is essential to comprehend the various industrial applications of thermochromic materials. Therefore, Table 11 provides a comprehensive illustration of their applicability across several sectors, highlighting the advantages they offer based on previous research and a variety of industrial conditions.

**Figure 12:**
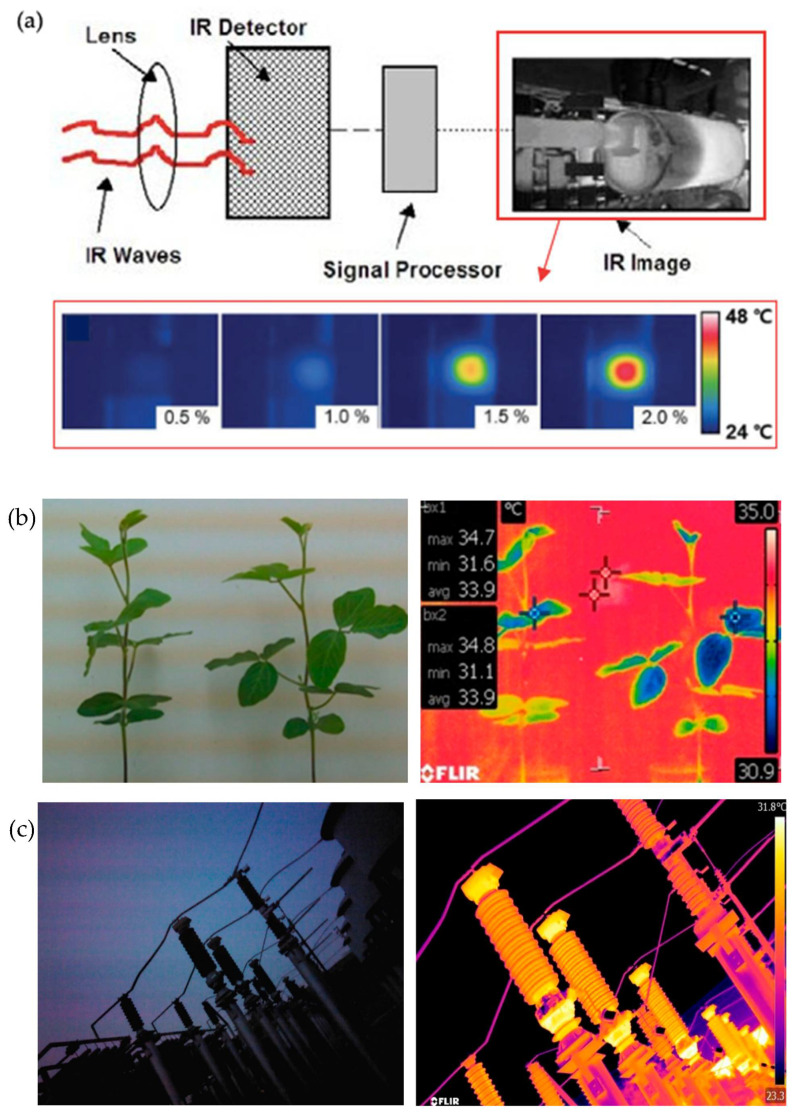
Thermal mapping of the thermal imaging technique emphasizes the thermochromic material and aids in thermal visualization. (**a**) Characteristics of thermochromic, crack-based sensors—IR camera images of each strain showing the thermal distribution on the sensor [213,214]. (**b**) Thermal images of soybean plants [215]. (**c**) Thermal images of power substation equipment [216].

Thermochromic materials have also been used in chemical engineering to help visualise the distribution of liquid temperature during microwave heating, which guarantees even heating, accurate reactions, and increased energy efficiency. For instance, reversible thermochromic microcapsules (RTMs) are used in paper manufacturing as a means of improving colour and in coatings in the wood coating industry. In the meantime, temperature-responsive microcapsules are used in electrical equipment to provide real-time monitoring and hotspot localization by allowing insulating materials to respond to extreme external temperatures [212]. Furthermore, Figure 12 depicts the significance of thermal mapping in thermochromic material applications through thermal imaging techniques, where it displays examples such as crack-based sensor thermal behaviour, thermal images of plants, and power substation equipment.

### 3.5. Health and Medical Device Application

Thermochromic materials in health and medical device applications offer significant promise for revolutionising healthcare through temperature monitoring, health parameter tracking, and innovative advancements. The characterization of these materials focuses on their sensitivity, precision in temperature monitoring, and biocompatibility evaluation. Evaluating their safety and compatibility with biological systems is crucial for their application in medical devices. Integrating thermochromic materials into wearable sensors and fabrics enables real-time monitoring of vital health parameters, fostering the development of smart clothing for medical purposes [217,218].

**Figure 13:**
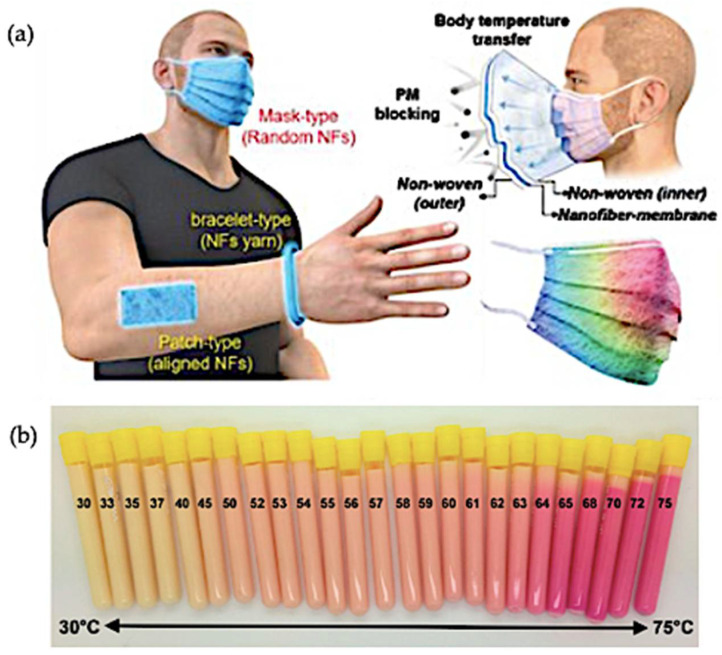
Innovative thermochromic material in health and medical device applications. (**a**) Fabrication process of reversible thermochromic nanofibrous membrane—potential applications in respiratory masks, patches, and bracelets [85,90]. (**b**) Development of tissue-mimicking thermochromic phantoms for MRI-imageable and HIFU-compatible temperature sensing [219,220,221].

Recent advancements in thermochromic sensing technologies showcase remarkable progress. Alam et al. [161] introduced polymer-based optical fibres embedded with thermochromic pigments, enabling precise temperature sensing through reversible spectral changes. Figure 13a illustrates these fibres’ structure and their response to temperature shifts, offering sensitivity and strain-sensing capabilities for expanded applications. Eranki et al. [219] developed tissue-mimicking thermochromic phantoms (TMTCPs), featuring MRI-imageable and temperature-sensitive properties, crucial for assessing high-intensity focused ultrasound (HIFU) during procedures. Figure 13b details the design of these phantoms, offering HIFU-compatible temperature sensing without invasive techniques, ensuring safe and efficient evaluation methods for medical procedures.

The recent advancements, as present in Table 12, underscore the concentrated efforts in tailoring polymer structures to enhance the flexibility, sensitivity, and durability of thermochromic materials used in medical devices. Innovations in polymer compositions aim to create adaptable and more sensitive medical devices, ensuring prolonged usage with increased patient comfort. Nanoparticle size significantly influences the performance of thermochromic materials, with recent developments focusing on precise control to impact sensing ranges, optimize optical performance, and mitigate potential toxicity concerns. Encapsulation methods play a crucial role in ensuring the reliability of these materials in medical devices, aiming for stability, safety, and faster response times. Surface modifications are being utilized to enhance adhesion properties and biocompatibility, enhance material stability within medical devices, and minimise adverse reactions. Innovative fabrication techniques ensure cost-effectiveness and scalability, meeting the rising demand without compromising quality.

Moreover, the information presented in Table 13 demonstrates significant progress in utilizing thermochromic materials across diverse medical applications. These materials have played pivotal roles, from enhancing accuracy and safety in medical imaging and thermal ablation techniques to enabling remote health monitoring and the potential development of smart medical clothing. Integration into optical fibre sensors offers crucial data for patient care and diagnostics, while 3D-printed polymer fibres with thermochromic properties enable tuneable sensing capabilities with flexibility and affordability.

### 3.6. Textiles and Fabric Applications

Integrating reversible thermochromic materials into textiles signifies a ground-breaking shift in heat detection and comfort enhancement, although it comes with durability and stability challenges. Recent studies by Zhang et al. [80] showcased inventive thermoresponsive dye systems enclosed in nanoparticles, yielding vibrant colour changes and strong fluorescence triggered by heat. Tözüm et al. [117] and Sahebkar et al. [165] have streamlined fabrication processes, enhancing the accessibility and cost-effectiveness of thermochromic fibres. These studies explored microencapsulation techniques, augmenting reversible colour changes, and thermal regulation crucial for adaptive clothing and protective wear [75,165].

**Figure 14:**
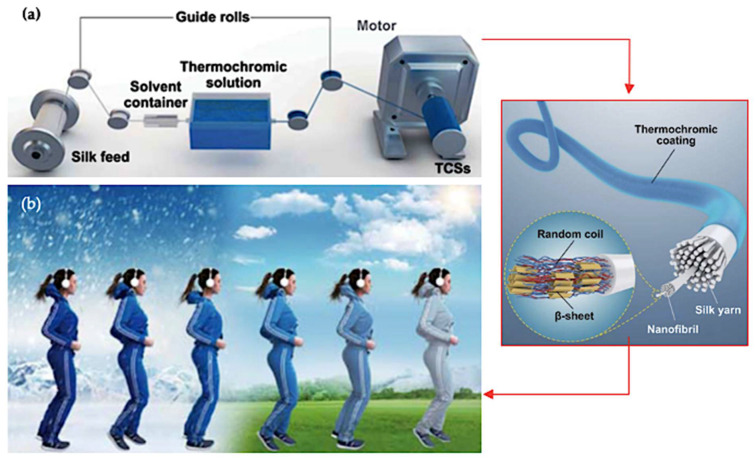
Innovations in thermochromic silk fabric technology. (**a**) Schematic diagram of process to produce TCSs using a continuous spinning device [77]. (**b**) Transition colour thermochromic fabrics effect in response to environment temperature [77].

Incorporating composite materials and microcapsules into thermochromic textiles, as highlighted by Yi et al. [78] and Geng et al. [79], enables dynamic responses to temperature fluctuations. These findings emphasise the stability of thermochromic features under extreme conditions, ensuring durability and reliability. Christie’s [147] research emphasises understanding the molecular mechanisms of thermochromic materials, which is crucial not only in textiles but also across various industries. Figure 14 illustrates a scalable approach introduced by Wang et al. [77] for producing thermochromic-coated silks (TCSs), unlocking applications in smart textiles, wearable devices, flexible displays, and human–machine interfaces.

**Figure 15:**
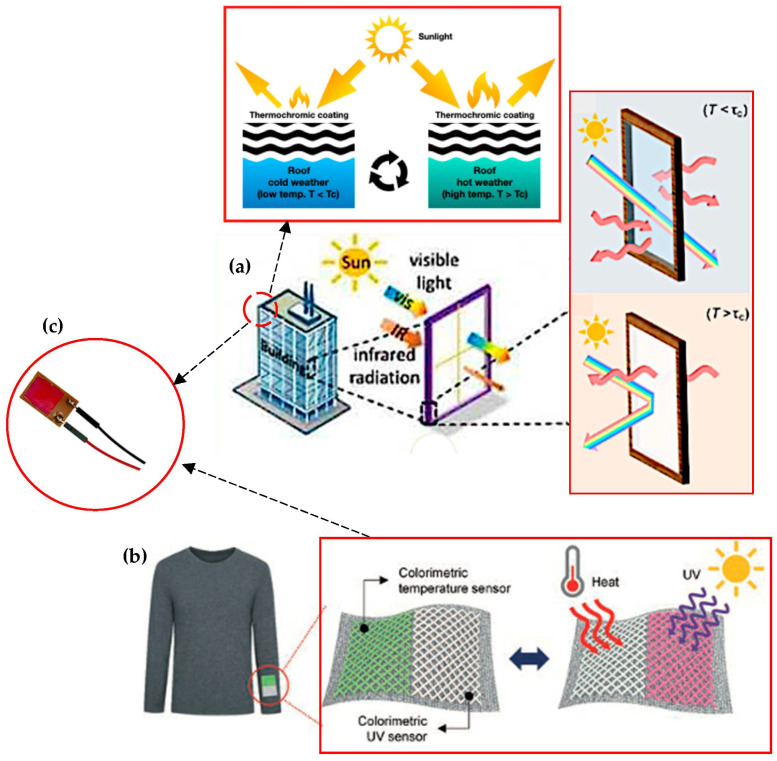
Versatile applications of thermochromic materials for smart application technology. (**a**) Hydrophobic roof coating and smart window module from thermochromic material [138,235]. (**b**) Calorimetric temperature and UV sensors applied by weaving thermochromic materials [236]. (**c**) Highly thermochromic temperature sensor membrane in fire detector sensor and temperature detection application [191].

Figure 15 illustrates the manifold applications of reversible thermochromic materials, spanning smart coatings, wearable sensors, energy management, and architecture. This innovative method bridges the gap between lab research and industry, driving functional textile advancements towards commercialization. Table 14 demonstrates the manifold benefits of integrating thermochromic materials into textiles. These materials enhance smart clothing by effectively regulating heat, improving comfort, and offering dynamic colour changes. Beyond fashion, these advancements play a pivotal role in temperature monitoring and comfort assurance. Adaptive fabrics enable real-time temperature tracking while enhancing comfort and aesthetics through dynamic colour alterations based on body temperature variations. Moreover, thermochromic materials boost safety in thermal-protective clothing by signalling dangerous temperatures through colour changes. Dynamic textile displays not only offer visual appeal but also adjust fabric aesthetics in response to temperature shifts.

## 4. Advantage and Challenges of Reversible Thermochromic Materials

This section explores the advantages and challenges posed by reversible thermochromic materials. Reversible thermochromic materials find wide-ranging applications, aiding in temperature monitoring and dynamic colour changes, such as in smart windows. However, maximising their benefits requires addressing challenges. Accurate calibration, maintaining colour stability, and preventing fading is critical. Table 15 summarises the strengths and limitations of reversible thermochromic materials. Despite their considerable advantages, overcoming stability, durability, and compatibility issues is essential across various industries.

### 4.1. Advancements in Material Design

Recent studies in reversible thermochromic materials are propelling their evolution for diverse applications. Researchers are dedicated to refining existing materials and developing new ones to enhance stability, durability, compatibility, and colour-changing properties. Breakthroughs include the creation of functional polymers with mechanochromic traits, suited for wearable electronic skin, smart screens, and anti-counterfeiting devices [243]. Geng et al.’s [184] work on reversible thermochromic microencapsulated phase change materials (TC-MPCMs) demonstrates exceptional thermal energy storage abilities via meticulous research on shell materials and encapsulation parameters.

### 4.2. Integration into IoT and Energy Applications

Reversible thermochromic materials integrated into IoT platforms are revolutionising temperature monitoring in healthcare, smart cities, and robotics [244,245]. Shi et al. [246] pioneering work on smart-textile-integrated microelectronic systems (STIMES) embeds sensors, displays, and energy harvesters into functional textile devices. Thummavichai et al.’s [152] exploration of tungsten oxide (WOx) nanomaterials drives innovation in smart windows, solar–thermal coatings, and gas–chromic hydrogen sensors. Ke et al. [160] explore adaptable smart window designs inspired by cephalopod skin, utilising vanadium dioxide (VO_2_) nanoparticles for improved energy efficiency.

### 4.3. Cost-Effectiveness and Versatility of Reversible Thermochromic Materials

Reversible thermochromic materials advance temperature monitoring by offering clear, easy-to-read displays of temperature changes for immediate recognition. They are a cost-effective alternative to complex electronic systems, suitable for various uses. These materials are highly adaptable, allowing customization for specific temperature ranges and colour changes. They seamlessly integrate into different surfaces, labels, coatings, and fabrics, boosting their versatility. However, challenges include the need for precise calibration and accurate reference temperatures for effective monitoring. Some materials may vary in sensitivity and response time, impacting real-time monitoring. Thus, ensuring stability and resistance to external factors is also a challenge, with impacts on overall performance (Table 16).

## 5. Future Directions and Recommendation

The potential of reversible thermochromic materials spans various applications, prompting a focus on stability enhancement, expanded temperature ranges, and feature enrichment. Ongoing research aims to achieve high-resolution monitoring, create tailored materials, and integrate these materials into smart systems. Innovation in fabrication methods and exploration of eco-friendly materials are also critical aspects. Emerging areas of interest include biomedicine, wearable tech, energy harvesting, photonics, and AI-driven data analytics. While offering numerous opportunities, ensuring durability and stability for extended use is crucial. This necessitates exploration into novel materials, protective coatings, and encapsulation techniques.

- Diverse application

Reversible thermochromic materials find applications in a wide array of fields. Christie [147] explored the potential of chromic textiles, discussing ongoing research trends driving technical and smart textile applications [252]. Mehta et al. [3] proposed a simple, cost-effective thermochromic temperature sensor, emphasising reusability and longevity for visual temperature monitoring without intricate circuitry.

b. Micro and nanofabrication techniques

Precise temperature mapping using micro- and nanofabrication techniques is crucial for specific industries. Advanced vanadium-oxide-based thin films in spacecraft thermal management offer reversible thermochromic and electrochromic properties vital for micro-satellite thermal regulation. Addressing challenges in commercial applications drives the need for further development and demonstrates the transformative potential of intelligent food packaging that can detect environmental changes and product conditions to enhance safety and reduce supply chain losses [253].

c. Environmental sustainability

The future of reversible thermochromic materials aligns with eco-friendly practices and innovative synthesis. Existing research emphasises smart, green interfaces using recyclable materials and sustainable production methods to reduce resource use. In addition, Kular et al. [163] explored the use of thermochromic inks on eco-friendly label stock derived from recycled materials. Additionally, a greener synthesis method, using a recyclable choline hydroxide catalyst, demonstrates the utility of sustainable approaches in reversible thermochromic material development. These eco-friendly strategies promise a greener future for reversible thermochromic materials.

## 6. Conclusions

In recent years, reversible thermochromic polymer nanocomposites have garnered increasing attention for their application in heat detection systems. This comprehensive review thoroughly examined the complexities associated with integrating these polymers into nanocomposite systems, assessing both their advantages and challenges. Among the synthetises processes of reversible thermochromic, solution-based synthesis and in situ polymerization are two most common techniques. Solution-based synthesis methods offer advantages such as scalability and control over material properties. By adjusting parameters such as solvent composition and polymer concentration, researchers can tailor the properties of reversible thermochromic polymer nanocomposites to suit specific applications. Additionally, solution-based synthesis allows for the incorporation of functional additives to enhance the performance of the nanocomposites. In situ polymerization techniques enable the direct synthesis of polymer nanocomposites within the desired matrix, facilitating better dispersion of nanoparticles and improved interfacial adhesion. This approach offers advantages in terms of process efficiency and control over material properties. The intrinsic ability of these materials to change colour in response to temperature fluctuations renders them highly promising for heat detection technologies. For instance, in building insulation, while these polymers can aid in identifying areas of heat loss or inadequate insulation, their restricted colour range may impede precise temperature detection. Moreover, their dependency on external stimuli like electricity or UV light can complicate implementation and escalate expenses. Addressing these challenges is paramount to fully unlocking the potential of reversible thermochromic polymer nanocomposites in heat detection systems. Continuous research efforts aimed at expanding colour options and reducing reliance on external triggers present promising avenues for enhancing their efficacy. Reversible thermochromic materials are highly advantages for applications such as building insulation. In this instance, reversible thermochromic polymer nanocomposites offer significant potential for improving building insulation by enabling the detection of areas with heat loss or inadequate insulation. However, the limited colour range of these polymers poses a challenge to precise temperature detection. Future research should focus on developing polymers with a broader colour palette to enhance their applicability in building insulation systems. Additionally, in industrial processes, reversible thermochromic polymer nanocomposites can play a crucial role in monitoring temperature changes and optimizing energy usage. However, their reliance on external triggers such as electricity or UV light may hinder their integration into industrial settings. Preferred synthesis methods should prioritize the development of stimuli-responsive polymers to mitigate dependence on external triggers and improve their suitability for industrial applications.

Despite their numerous advantages, challenges persist, including the need for precise calibration, stability maintenance, and compatibility with various substrates. Overcoming these hurdles demands further research and development to enhance stability, durability, and compatibility, thereby broadening their adoption. Looking ahead, reversible thermochromic materials hold the promise of innovative temperature monitoring and control solutions. Additionally, reversible thermochromic polymer nanocomposites hold immense potential for heat detection systems, addressing challenges related to limited colour options and dependence on external triggers, which is imperative for their widespread adoption. Preferred synthesis methods such as solution-based synthesis and in situ polymerization offer avenues for optimizing the properties of these nanocomposites. Future research should prioritize expanding colour options and reducing reliance on external triggers to enhance the effectiveness and practicality of reversible thermochromic polymer nanocomposites in heat detection applications.

## Figures and Tables

**Table 1:** Summary of chromic phenomena and main technologies.

Chromic Phenomena/Stimulus				
Stimulated Colour Change	Absorption of Light and Energy Transfer	Absorption of Energy and Emission of Colour	Absorption/Reflection	Manipulation of Light
Photochromism Thermochromism Electrochromism Ionochromism Solvatochromism Vaporchromism Gasochromism Mechanochromism Excitonic Coupling Plasmonic Coupling	Photothermal Photoelectricity Photoconduction Photochemistry Photoconversion	Phosphorescence Fluorescence Biofluorescence Chemiluminescence Bioluminescence Electroluminescence Electrochemiluminescence Triboluminescence	Classical colouration by organic dyes Organic and inorganic pigments natural colourants	Refraction Diffraction Reflection Scattering Interference Amplification Nonlinear optics
Technologies Based on Chromic Phenomena				
Ophthalmic Safety and security Thermal printing Smart window and mirror Analysis and sensing	Optical data storage Laser printing and photocopying Photopolymerization Photomedicine Solar energy and photosynthesis	Display monitor Lighting Florescence colouration Dye lasers Chemical analysis Biomedical analysis Genomics Proteomics	Colouration of textiles Paint, plastic, paper, and leather Hair and cosmetic Food and beverages Photography Digital printing and imaging	Display Optical data storage Holography Effect colouration Laser diodes Optoelectronic Photonics

**Table 2:** Classification of thermochromic property studies.

Classification	Property	Trigger Mechanism	Application/Technology	References
Irreversible	Chemical change	Chemical reaction Irreversible colours change upon heating	Development of irreversible thermochromic dyes for temperature-sensitive labels	[54,62,71]
			Utilization of irreversible thermochromic materials in food safety indicators	[71,72,74]
	Phase transition	Solid to liquid transition Irreversible colours change upon heating	Study on irreversible phase transition thermochromic material in polymer composites	[19,52,67]
			Utilization of phase transition thermochromic material in clothing industry	[77,78,79,80,81]
Reversible	Molecular movement	Conformational changes Revisable colour change based on molecular rearrangement	Development of smart windows using revisable thermochromic coating	[26,58,77]
			Research on revisable thermochromic ink for temperature-sensitive labels	[79,80,81,82]
	Particle dispersion	Aggregation/dispersion of nanoparticle Reversible change in nanoparticle arrangement leading to colour change	Investigation of revisable thermochromic polymer nanocomposites for energy-efficient buildings	[38,78,83]
			Utilization of reversible nanoparticle-based thermochromic material in energy-saving glass	[37,38,77]

**Table 3:** Thermochromic mechanisms, applications, and future research in reversible nanocomposites.

Mechanism	Description	Parameters	Function	Future Research Development	References
Crystal Transition	It involves lattice displacement in metal ion compounds due to temperature changes, altering crystal structure and, consequently, material colour.	Critical transition temperature: 75 °C Crystal lattice expansion rate: 0.1 nm/°C	Smart coatings Cool roofs Automotive paints Sensors	Investigate advanced crystal transition processes for improved colour adaptation in extreme temperature ranges.	[84,85,86,87,88,89,90]
Ligand Geometry	Alterations in molecular structure and geometry, particularly in materials containing Cr^3+^ ions, lead to colour changes with temperature fluctuations.	Ion spacing: 2.5 Å Geometry alteration rate: 0.02 °C^−1^	Utilised in smart packaging for perishable goods Indicating temperature changes or product freshness for quality maintenance	Explore the design of more sensitive and responsive materials for precise temperature indications in diverse environmental conditions.	[91,92,93]
Coordination Number	Influences colour changes in inorganic salts due to the presence or loss of crystal water molecules with temperature variations.	Coordination geometry: Orthogonal Crystal water content: 4 g/mol Moisture absorption rate: 15%	Applications in humidity sensors Smart packaging Chemical monitoring systems	Research on materials that can adapt to specific environmental factors, offering wider applications in diverse industries.	[46,76,94]
Liquid Crystal	It involves changes in the wavelength of reflected light due to temperature-induced variations in the pitch of spiral configurations.	Spiral pitch variation: 10 μm Light wavelength modulation rate: 5 nm/°C	Smart textiles for clothing and sensors in industrial settings Changing colour based on body temperature or equipment heat	Develop materials with tailored responses to temperature variations for advanced applications in wearables and industrial sensors.	[95]

**Table 4:** Schematic of synthesis methods for reversible thermochromic materials.

Method	Key Features	Advantages	Limitations	References
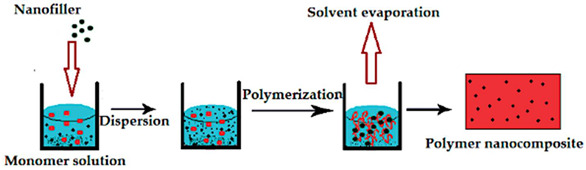				
In situ polymerization	A polymer matrix forms simultaneously with the incorporation of thermochromic components.	Precise control over component distribution.	Suitable for thermoplastic polymers. Requires careful solvent selection and processing to avoid issues.	[103,105,106,107,108,109,110,111]
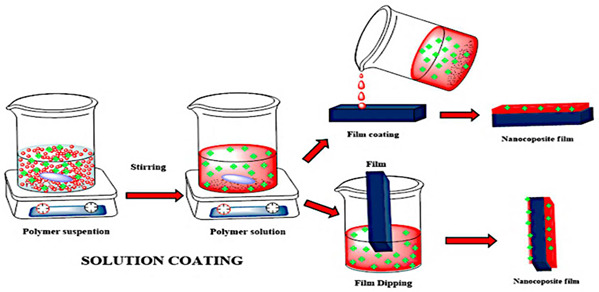				
Solution mixing	The thermochromic chemicals and polymer matrix are dissolved in a solvent.	Simplicity and scalability.	Limited to thermoplastic polymers. Challenges related to uniform distribution.	[112,113,114]
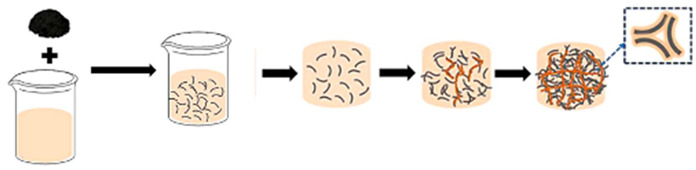				
Melt blending	Thermochromic compounds are combined with a polymer matrix at elevated temperatures.	Suitable for thermoplastic polymers.	Compatible with common polymer processing methods. Difficulties with uniform dispersion. Possible compound degradation during processing.	[115,116,117,118]
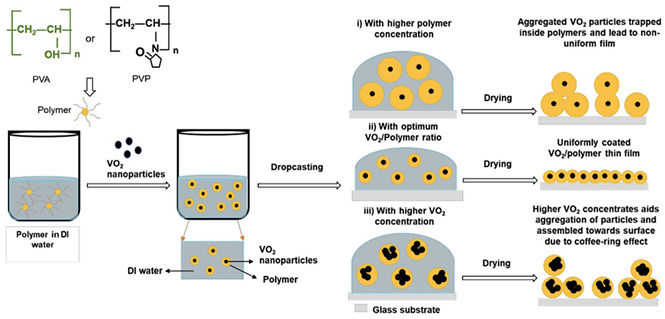				
Nanoparticle encapsulation	Thermochromic nanoparticles are incorporated into a polymer matrix.	Precise control over nanoparticle characteristics Tailored thermochromic behaviour.	May only work with specific systems. Issues with nanoparticle stability and dispersion.	[119,120,121]
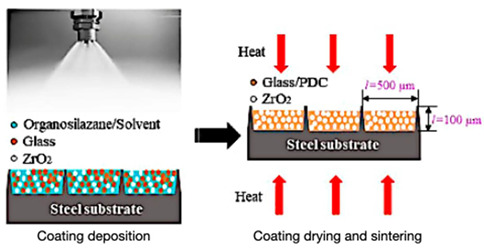				
Coating and impregnation	Addition of a thermochromic layer to a substrate or infusion of a substrate with thermochromic chemicals.	Applicable to various substrates. Compatibility with different methods.	Challenges with uniform coverage on complex surfaces. Potential durability issues under harsh conditions.	[122,123,124,125]

**Table 5:** Characteristics and applications of thermochromic material in heat detection systems.

Characterisation	Description	References
Solar Energy Regulation	Dynamically modulate solar energy transmission through smart windows. Regulate their transparency or reflectivity in response to temperature changes, helping to control heat gain from sunlight.	[126,127,128]
Temperature responsiveness	Variation their optical properties with temperature variations—reducing the amount of sunlight entering a building—becomes more transparent as the temperature decreases.	[129]
Visible–Near Infrared Regulation	Effectively regulate the transmission of visible and near-infrared light—control of both visible light and heat from the sun. Contributing to energy-efficient building temperature management.	[130,131,132,133,134,135,136,137]
Energy Savings	By regulating the amount of solar energy entering a building, thermochromic smart windows contribute to energy savings by reducing the need for air conditioning or heating—maintain a comfortable indoor environment.	[130,131,132,133,138]
Colour-Changing Properties	Change colour transition between different states and utilised for aesthetic purposes or as a visual indicator of the window’s current state.	[40,43,54,139]
Quick Response	Many thermochromic materials have rapid response times—allowing them to adapt quickly to changing environmental conditions—efficient energy management	[41,74,80,119,140]
Durability	Advances in material science have led to the development of durable thermochromic coatings that can withstand long-term exposure to outdoor conditions without degradation.	[135,136,137]
Integration	Thermochromic materials can be integrated into various window types, including glass and transparent films, making them versatile for use in both new construction and retrofitting existing buildings.	[141,142,143,144,145]
Potential for Energy Storage	Some thermochromic materials not only regulate solar energy but also have the capacity to absorb and store thermal energy, which can be released when needed for heating or cooling.	[77,141,146,147,148]
Application in Sustainable Architecture	Thermochromic smart windows play a vital role in sustainable and energy-efficient building designs. Contribute to reduced energy consumption, improved comfort, and a lower environmental impact.	[42,130,149,150]

**Table 6:** Summary of characterization techniques for reversible thermochromic materials.

Characterization Technique	Purpose/Information	Notable Findings and Applications	References
Spectroscopic Techniques (UV–Vis and FTIR spectroscopy)	Analyse optical properties Detect colour changes	UV–Vis detects colour changes based on wavelength, while FTIR reveals structural modifications and bonding changes. This is important for understanding how these materials behave optically.	[70,151,152]
Microscopic Techniques (SEM and TEM)	Investigate morphological changes through particle size and size distribution	High-resolution imaging allows detailed analysis of surface characteristics and particle distribution. Useful for assessing morphological changes and surface characteristics.	[47,50]
Thermal Analysis Techniques (DSC and TGA)	Measure heat flow Phase transitions. Enthalpy changes Determine thermal stability	DSC provides phase transition temperatures and enthalpy changes, while TGA determines thermal stability and breakdown temperatures. Essential for evaluating material compatibility at different temperatures.	[103]
Dynamic Mechanical Analysis (DMA)	Assess viscoelastic behaviour Glass transition temperatures Mechanical stability	It helps understand how these materials behave under different temperature and frequency conditions.	[153,154,155]
Mechanical Testing Methods (Tensile Testing)	Material strength Elongation Flexibility Hardness Adhesion	Evaluates the overall mechanical performance, robustness, and wear resistance of the material.	[156,157,158,159,160,161]
Surface Analysis Techniques (AFM and XPS)	Surface morphology Roughness Chemical composition	Provides insights into surface interactions. Crucial for assessing long-term stability and colour-changing abilities.	[54,162,163,164,165]

**Table 7:** Overview of techniques and their roles in assessing thermochromic nanocomposites material.

Technique	Purpose/Role	Parameter/Value	References
Zeta Potential Analysis	Understanding particle interactions, emulsions, and suspensions are important for comprehending behaviour.	Zeta potential value (−20 mV), indicating particle stability or interaction strength.	[109,167,168]
Polarised Optical Microscopy	Visual assessment is critical for unravelling optical intricacies, especially anisotropic behaviour.	Observing birefringence patterns indicating anisotropic behaviour.	[41,151,169,170]
Chemical Analysis	FT-IR and X-ray diffraction techniques aid in unravelling chemical properties and interactions.	Identification of specific functional groups (e.g., -OH or -COOH) in FT-IR spectra.	[160,161]
Topographical Analysis	SEM and TEM evaluations offer insights into morphology and surface characteristics.	Measurement of nanoparticle size (e.g., 50 nm) or surface roughness observed in SEM or TEM images.	[146,162,163,164]

**Table 8:** Applications and utilization of thermochromic nanomaterial in various fields.

Application	Thermochromic Development	Performance Function	Previous and Current Research	References
Smart window	Window coatings: transparency and reflectivity based on temperature	Energy savings Environmental impact	Vanadium oxide nanofilms for energy-efficient windows	[40,172,184,185]
Temperature sensors	Temperature-sensitive paints coatings change colour with temperature variation	Visual Indication	Industrial machinery hotspot detection with temperature-sensitive paints	[174,186]
Medical application	Medical device coatings Monitoring body temperature or diagnostics	Non-invasive patient comfort	Wearable thermochromic patches for temperature monitoring	[105,178]
Thermal insulation	Building insulation materials reflect or absorb heat based on temperature	Improved insulation and energy efficiency	Insulating materials for buildings that adapt to temperature changes	[26,38,155]
Food quality and safety	Food packaging Change colour if the temperature exceeds a safe range	Food safety and quality assurance	Thermochromic labels on perishable goods packaging	[72,142,187]
Fire safety	Fire-resistant coatings change properties when exposed to high temperatures	Early detection improves safety	Fire-resistant coatings with thermochromic additives	[150,188,189,190]

**Table 10:** Characteristic of thermochromic material in food packaging.

Thermochromic Material	Application	Advantages	Limitations	References
Thermochromic Ink	Labels and Foils	Visual temperature indication Quality and shelf-life indicators Enhanced communication with consumers	Requires calibration Potential health hazards Compatibility with food contact	[62,71,72,79]
Photochromic and thermochromic colourants	Food Packaging	Temperature monitoring Quality control Regulatory compliance	Longevity Adhesion Chemical or moisture resistance	[40,62,156]
Microcapsules	Ink and film materials Polymers engineered to exhibit thermochromism	Improved quality control Visual temperature indication Potential use in food packaging	-	[47,48,50,63]

**Table 11:** Applications of thermochromic materials in industrial equipment and manufacturing.

Industrial Area	Key Use Thermochromic Materials	Benefits and Significance	References
Chemical Engineering	In situ imaging of liquid temperature distribution during microwave heating	Addresses uneven heating Improves reaction precision Enhances energy efficiency	[149,208]
Paper Manufacturing	Incorporation of reversible thermochromic microcapsules (RTM) to create secure and counterfeit-resistant materials. Investigation into RTM retention in pulp for improved material security and anti-counterfeiting features	Enhances security and anti-counterfeiting measures in various industrial applications	[158,159,209]
Wood Coating Industry	Utilising reversible thermochromic aqueous coatings to control colour differences and gloss of coatings The optimisation of the coating process has potential applications in furniture engineering	Offers control over coating quality and creating intelligent wood coatings	[81,210]
Electrical Equipment	Development of temperature-responsive microcapsules to enable insulating materials from excessive external temperature Colour changes in response to temperature variations and localization of hotspots. Enhances real-time monitoring and safety of electrical and electronic devices	Improves the safety and functionality of electrical equipment and enhances temperature monitoring.	[105,211]

**Table 12:** Chemical engineering insight on thermochromic material in medical devices.

Chemical Composition	Role in Biocompatibility and Material Functionality	Element	References
Polymer structure	Influences material properties	Flexibility Sensitivity Durability	[62,67,103,222]
Nanoparticle size	Affects material characteristics	Sensing range Optical performance Toxicity	[105,115,223]
Encapsulation methods	Determines material reliability	Stability Safety Response time	[67,105]
Surface modifications	Influences material interaction	Adhesion properties Biocompatibility	[49]
Material fabrication	Determines production feasibility	Cost-effectiveness Scalability	[205]
Environmental impact	Understanding material performance	Behaviour in varying conditions	[151,223,224]

**Table 13:** The advantage and applications of thermochromic materials in health and medical device.

Medical Application Area	Advantage of Thermochromic Materials	Function	References
Medical Imaging	Direct visualisation and quantification of HIFU heating Enhanced accuracy and safety during medical procedures Accurate tissue-mimicking properties for evaluating thermal therapy equipment	Accuracy in HIFU heating Safety in medical procedures Tissue-mimicking properties	[115,225,226,227]
Thermal Ablation Techniques	Improved quality and accuracy in procedures like laser and microwave ablation Enhanced medical device performance and patient safety	Quality and accuracy Device performance	[115,228]
Remote Health Monitoring	Real-time monitoring of vital health parameters Potential for smart clothing for medical applications	Real-time monitoring Smart clothing potential	[75,229,230]
Optical Fibre Sensors	Simultaneous measurement of temperature and humidity in healthcare settings Vital information for patient care and diagnostics	Temperature and humidity Patient care and diagnostics	[231,232]
3D-Printed Polymer Fibres	Tuneable strain and temperature sensing in biomedical and healthcare applications Flexibility, reusability, and affordability	Strain and temperature Flexibility and reusability	[161,233,234]

**Table 14:** The use of thermochromic materials in textiles and fabric applications.

Application	Advantage of Thermochromic Materials	Function	Temperature Range (°C)	Colour Change Threshold (°C)	Sensitive to Temperature Changes	References
Smart apparel	Offers temperature-responsive colour changes; Enhances thermal comfort and heat regulation in clothing.	Enhancing thermal comfort and heat regulation in clothing	20–40 °C	±2 °C	High	[52,207,237]
Adaptive textiles	Provides dynamic colour changes based on body temperature fluctuations; Offers improved comfort and aesthetics.	Offering improved comfort and aesthetics	25–50 °C	±3 °C	Moderate	[75,134,156]
Thermal protective garments	Changes colour in response to temperature changes; Indicates potential exposure to hazardous temperatures.	Indicating potential exposure to hazardous temperatures	25–50 °C	±3 °C	Moderate	[105,155]
Dynamic textile displays	Allows for fabric displays that change colour or patterns based on environmental temperature changes.	Aesthetic display Temperature indication	20–40 °C	±2 °C	High	[52,61,75,115]
Wearable devices	Integration of thermochromic materials in clothing for wearable technology; Indicates changes in body temperature or environmental conditions.	Indicating changes in body temperature or environmental conditions	15–35 °C	±1.5 °C	High	[59,75,163,238]
Health monitoring	Real-time monitoring of vital health parameters.	Real-time health monitoring	15–35 °C	±1.5 °C	High	[239,240,241]
Industrial uses	Temperature indication for industrial equipment and processes.	Process optimization	30–45 °C	±2.5 °C	Low	[105,149,164]
Environmental sensors	Temperature sensing for environmental monitoring.	Environmental monitoring	30–45 °C	±2.5 °C	Low	[49,72,147]

**Table 15:** Advantage and challenges of reversible thermochromic materials.

Aspect	Advantage	Challenge	References
Cost	Cost-effective compared to complex electronic monitoring	Accurate calibration is needed for a successful temperature display	[62,112,122]
Versatility	Versatile application on various surfaces and materials	Changes in sensitivity and response time can affect monitoring	[84,162,205]
Ease of use	Simple and straightforward temperature indication	Ensuring long-term stability and preventing colour fading	[72,149,242]
Customizability	Can be tailored to different applications and industries	Maintaining resilience to external factors	[60,155]
Applications	Wide range of potential applications in diverse industries (surfaces, labels, coatings, and fabrics)	Material compatibility with various substrates	[162,205,222]

**Table 16:** A comparison of the cost-effectiveness of reversible thermochromic materials to traditional electronic systems.

Factors	Reversible Thermochromic Materials	Traditional Systems (Electronic)	References
Initial setup costs	Lower	Higher	[164,232,241]
Maintenance expenses	Lower	Higher	[27,184,247,248]
Long-term reliability	Generally good	Subject to wear and tear	[184,242,247]
Energy consumption	Lower	Higher	[83,132,249]
Integration flexibility	High	limited	[140,184,247,250]
Customization possibilities	Flexible	Rigid	[140,184,247,250]
Scalability	Varies	Complex	[132,140,184,247]
Environmental impact	Eco-friendly	Electronic waste concern	[163,184,251]
Response time	Varies	Fast	[50,164]
Sensing range	Adaptable	Fixed	[184,191,247,250]

## Data Availability

No new data were created or analysed in this study. Data sharing is not applicable to this article.
